# Oral health and medical conditions among Amish children 

**DOI:** 10.4317/jced.53468

**Published:** 2017-03-01

**Authors:** Masahiro Heima, Marc-Allen Harrison, Peter Milgrom

**Affiliations:** 1 DDS, PhD, Assistant Professor, Department of Pediatric Dentistry, School of Dental Medicine, Case Western Reserve University; 2 BS, DMD, Department of Pediatric Dentistry, School of Dental Medicine, Case Western Reserve University; 3 DDS, Professor, Northwest Center to Reduce Oral Health Disparities, University of Washington

## Abstract

Background: The Amish are a growing population who live a traditional, rural way of life, which makes them less accepting of modernism. Most Amish live in poverty and are detached from modern health care. In addition, the recent change of their lifestyle has been reported, such as consuming a nontraditional diet and the usage of electronic devices. As a result, their lifestyle change may have impacted their oral health. However, since only a single report about oral health among Amish children has been published approximately three decades ago it has not yet been updated. This study describes oral health among Amish children and their medical conditions during visits to a mobile dental unit (MDU). Material and Methods: The dental records of all patients (N=216) who visited a mobile dental unit were reviewed, which covers 1 year from May 20, 2011, the first date of service. The following factors were taken into consideration during the review process: parental perceptions of their children’s oral health care, dental care experiences, and general health information. Results: Fifty-four (27.8%) children, ages 3 to 17, have never received dental treatment before visiting the MDU; the average number of untreated decayed teeth was 6.8. In spite of this, most parents rated their children’s oral health as good or very good (87.7%). The high cost and long distance travel associated with routine, professional dental care makes it difficult for children to maintain good oral hygiene. Our analysis revealed that bleeding disorders were more prevalent among this gene pool compared to the nation at large; however, asthma was less common. Conclusions: There are oral and general health disparities among Amish children. There is a lack of awareness among Amish parents with regard to their children’s oral health.

** Key words:**Amish, child, dental caries, mobile health units.

## Introduction

The Amish are a growing group of Protestant Christians, and they are distinct from non-Amish by the simplicity of their lifestyle and detachment from the conveniences of modern technology, such as electricity and motorized machines (1). Although some Amish have begun to use modern medical therapy, others still strongly prefer alternative medicine, traditional remedies, homeopathic and natural treatments due to their religious practices and beliefs (2-5). For this reason, immunization rates among Amish children were low; 68% of parents indicated that their children have received at least one immunization; 86% of parents completed the vaccination exemption form for their children. A contributing factor to the low immunization rate among children is the fear that parents have with regard to the adverse effects of the vaccines, regardless of health care accessibility (6). Another essential factor affecting Amish health is marriage, which traditionally occurs only between members within their community. Consequently, many cases of rare genetic disorders, such as Cohen Syndrome, Ellis-van Creveld syndrome, have also been reported in the Amish communities (7).

Today, the Amish have adopted many aspects of modern society: adults often work outside of their communities, and shop at stores near their place of work. As a result, the Amish currently consume a more non-traditional diet (8-10). Although, societal and parental influences directly impact children’s oral health (11), current oral health of Amish children is poorly understood.

A single study in 1988 clinically examined 68 children under 17 years of age residing in one Amish community in Michigan (12). Although no radiographs were taken and convenience sampling was employed, the author concluded that Amish children had a surprisingly low dental caries prevalence in spite of inadequate oral health care knowledge. The report attributed the low rate of sugar diet. However, this circumstance might have changed and there is no updated report based on our knowledge .

Despite a growing number of Amish families using modern health care today, health disparities still exist, mainly due to their religious customs and beliefs.

At the request of this Amish community, our pediatric dentistry team operated a dental mobile unit (DMU) for 1 year starting in May 2011. Four days per month the DMU served the Amish community of Geauga County in Ohio. Our patient sample was primarily attained through local advertisements, newspapers and referrals from a medical clinic.

This report explains the oral and general health concerns among the Amish children who participated in the mobile dental unit.

## Material and Methods

-Study design: Retrospective chart review study. All charts from the first date of service, May 20, 2011, through May 19, 2012, from the Geauga County site were reviewed.

The state of Ohio is the most populated Amish location in the United of States and Geauga county is the second most Amish populated county in Ohio (1). In Geauga county, in the year 2012, the ratio of dentist to population was 1:1993; it was lower than the national weighted average which has a ratio of 1:1667 (13).

According to the 2012 United States Census Bureau, there were 22,997 people, age 0 to 17 and 903 births in Geauga County. The per capita income of Geauga county in 2011 was $51,165, and 12.3% of children under age 18 was living in poverty (14).

-Data elements: The oral and general health record information for each child was reviewed, which included information on each child’s parental education level, insurance, and home address. Parents completed a 5-item questionnaire addressing the following: “How long has it been since your child last visited the dentist? (within the past year, 1-3 years ago, more than 3 years ago, never have been, I don’t know)”; “During the past 12 months, was there a time when your child needed dental care, but could not get it at that time?” ; “What was the main reason the child couldn’t get care? (could not afford, dentist did not accept Medicaid/insurance, difficulty in getting the appointment, no way to get there, other reason, I don’t know)”; “Please describe the condition of your child’s teeth (very good, good, poor)”; “During the past 6 months did your child have a toothache? (yes, no, don’t know)? ” The Flesch-Kincaid Grade Reading Level of the questions was 3.5.

Standardized dental caries assessments, including tactile-visual examination and bite-wing radiographs were completed by pediatric dentists or residents, and recorded at the surface level on a tooth chart.

Outcomes were abstracted from the tooth chart: decayed primary teeth (dt), primary teeth with either decay or filling (dft), decayed permanent teeth (DT), and decayed, missing, or filled (DMFT) permanent teeth. We also abstracted general health information, which was above category 2 of the American Society of Anesthesiologists physical status classification system, which include “patients with mild systemic disease”, like Asthma (15).

Statistical analysis: Descriptive analysis was performed for Amish children and their parents in the areas of general health, dental accessibility and dental care. The Amish distance to travel from home to dental treatment was tracked using Google Maps (Google Inc., California, USA.) Outcomes were grouped by children’s ages: 3-5, 6-11, 12-15, and ≥16 years old. Tests regarding the differences of parents’ perception and the actual dental caries care index (dt + DT) were analyzed by using the analysis of variance (ANOVA) tests. All statistical analyses were conducted by IBM SPSS statistics 22.

The Institutional Review Board of our institute approved the research protocol.

## Results

-Characteristics of patients and parents: We reviewed 216 charts; six children’s charts were excluded from analyses because they were incomplete. The questionnaires were mainly completed by Amish mothers (94.8%). Most parents (184/185) did not complete high school; 25 parents did not answer this question. The mean (SD) age of the children was 10.32 (3.70) years with a range from 3 to 17; 44.9% were male. 18.5% (40) were above the category 2 physical status (medically compromised at least “mild systemic disease”) (15), including 11 (5.09%) with bleeding problems, and 13 (6.02%) had asthma.  Table 1 shows the distribution of general health, which the dentists encountered.

**Table 1:**
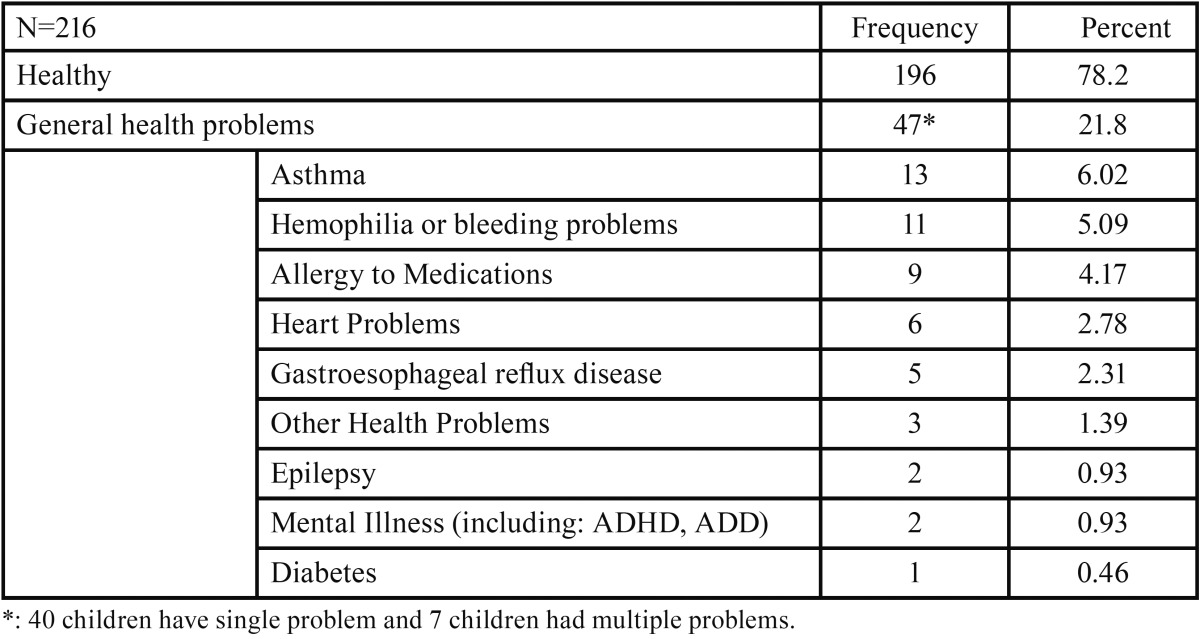
General Health Problems Reported by the Parents of Amish children.

-Oral health care service accessibility: Only 33.2% (66) children visited a dentist in the past year. About one-quarter (54/194, 27.8%) had never seen a dentist (mean (SD) age = 7.8 (3.4) years). The average (SD) travel distance was 10.3 (8.5) km; 25% traveled more than 13.9 km. Most were uninsured (86.6%) and were not enrolled in Medicaid, which is a governmental social health care program for families and individuals with limited resources, even though it was available to them. Almost a third (65/197, 31.0%) reported they could not get dental care for their children when they needed it in the past 12 months; among those, the most frequent reason was “I could not afford the dental visit” (42/65, 64.6%).

-Oral health:  Table 2 shows the dental health of Amish children. Only 11 children were caries free. The typical Amish child had 6.8 untreated decayed primary or permanent teeth and 36.2% of the parents (76/210) reported the child had a toothache in the last 6 months. Across age categories more than 88% of children have untreated decayed teeth. Nevertheless, most parents rated their children’s health as good or very good (139/159, 87.4%). Our clinical observation is that parents were not very concerned about their child’s teeth. We have provided many permanent tooth extractions and dentures for teen patients. An Amish girl told us “My mom and my sisters had a denture when they were teenagers. Many Amish lost their teeth when they were young.” 

**Table 2:**
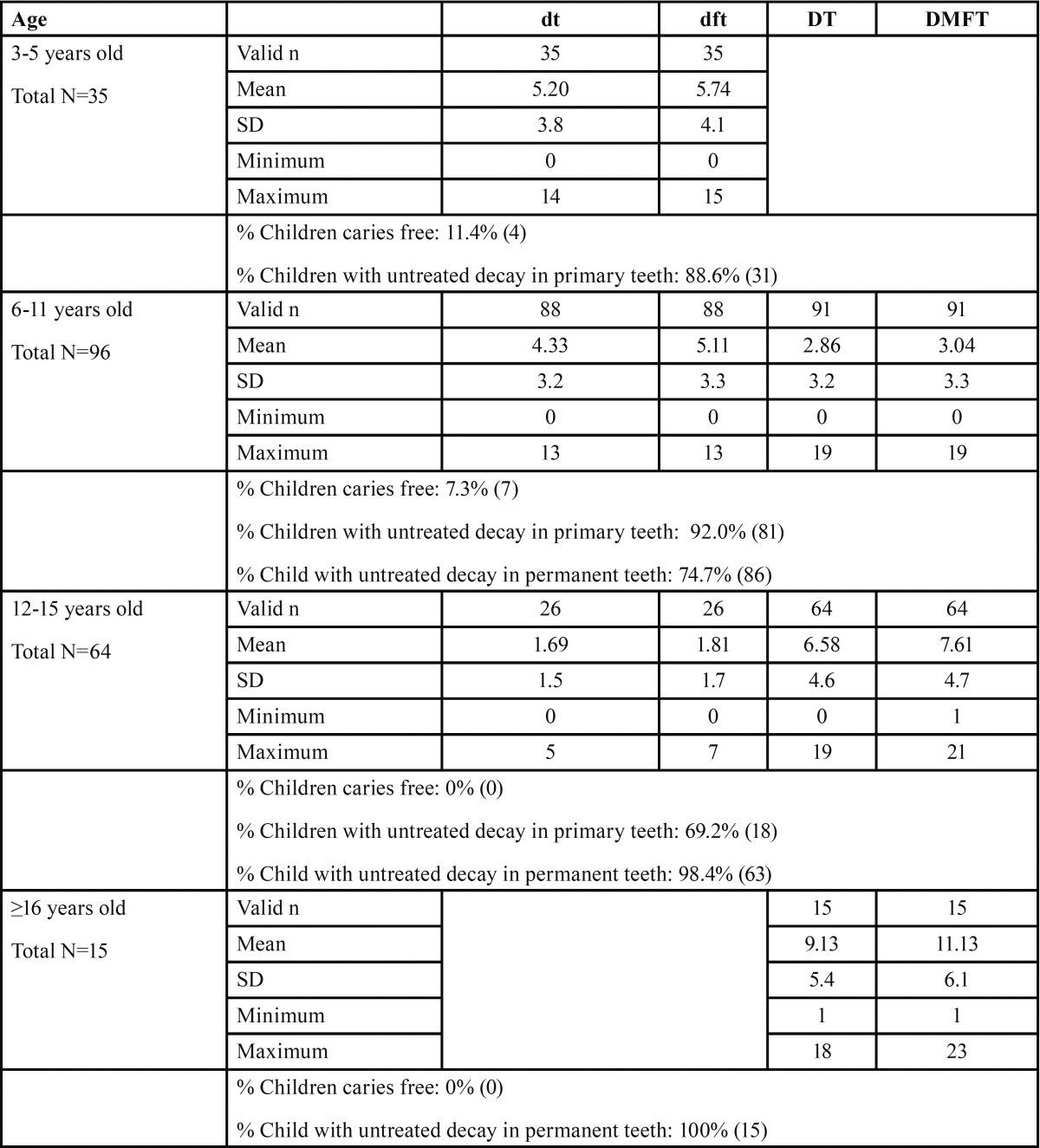
Oral Health of Amish children in Geauga County, Ohio seen by the mobile clinic (N=211).

-Parental perception of their children’s oral health and the number of untreated dental caries: The analysis of variance (ANOVA) was used to test the differences between children’s number of untreated decayed teeth and parental perception of their children’s oral health: very good (n=4)”, “good (n=135)”, and “poor (n=20)”.

The mean (SD) of children’s number of untreated decayed teeth were 1.25 (1.50), 6.28 (4.18) and 6.65 (3.44) respectively. It indicated a significantly different number of decayed teeth among the three parental perceptions (F=3.12, *p*=0.047) and Tukey HSD post-hoc multiple comparisons indicated “very good” group has significantly less caries than others (vs. “good” *p*=0.041, vs. “Poor” *p*=0.043). However, the number of decayed teeth between “Good” and “Poor” groups were very similar (*p*=0.924).

## Discussion

Although we gathered our data from patient charts, we found Amish children had high levels of untreated tooth decay. It was consistent with the disparities of children’s oral health seen in other rural communities in the U.S. These families must travel long distances by non-automobile transportation to seek care, and care is often not readily available because of their financial difficulties. Families use well water to drink and do not access fluoridated water. Although the recruitment of participants is different between convenient population and patients of the mobile dental unit, these clinical findings seemed in stark contrast to the 1988 report from a similar Amish community (12).

There are some possible reasons poor oral health exists among Amish patients: Amish are fatalistic (16) and do not view preventive care as a priority (17), which is consistent with reports that many Amish children are not vaccinated (6). Further, Amish are willing to suffer from their diseases and lean on the providence of God, and they believe God is the ultimate healer. Amish cultural norms eschew accepting governmental health supports (12), and as a result they are left with the option to pay expensive professional dental care fees out-of-pocket. Furthermore, we could not see any association between dental accessibility (travel distance) and the prevalence of Amish children’s dental caries in preliminary analyses. However, it is the fact that the families have to travel long distances for care. At last, parental education level and awareness levels of their child’s oral condition are important factors in children’s dental caries prevalence (18), however, we found many Amish parents were unaware of the actual state of their teeth.

We are not able to conclude that the high prevalence of dental caries in Amish children was caused by the life changes, poverty, failure to seek preventive care, changing diet, or selection bias. However, compared with the U.S. population as a whole, the caries (dft) prevalence for 3 to 5 year olds was 4 fold higher (88.6% in Amish vs. 20.48% in national data (19)) and similar to rates published for Alaska Native or American Indian children who live in non-fluoridated isolated villages, which are similar to the Amish community; the reported untreated dental caries prevalence among Alaska Native children with decayed primary teeth (dt) is 65% among 4 to 5 year olds. The rate of untreated teeth among older children was 54% among 6-8 year olds, 45% of children among 9-11 year olds (DT), 66% among 9-11 year olds (DT), and 68% among 12-15 year olds (DT) (20). Another report also showed 60.9-75.1% of 3-5 year old Alaska Native or American Indian children had tooth decay (21). Thus, the general magnitude of the rates is plausible.

We encountered more medically compromised Amish children at the mobile dental unit site for the Amish than usual. Many Amish children suffer from conditions from gene abnormalities (7). The rate of “bleeding problems,” such a von Willebrand disease and hemophilia B, were 5 fold higher (5.1 % in Amish vs. 1% in national data) (22,23). Dentists who care for Amish children may be the first health care provider to recognize a bleeding disorder. On the other hand, physicians who provide care of bleeding disorder might recognize poor oral health in their Amish patients. In contrast, the prevalence of asthma (6%) was lower than the national rate (2012 National Health Interview Survey Data, 14.3%), perhaps reflecting fewer environmental challenges (24).

This report provides a rare snapshot of concerns regarding oral health in a single Amish community: 1) there are notable disparities in dental access and oral health, and 2) high rates of major health problems among Amish children who visited the mobile dental unit. The next step in this study is to conduct a population based research in of Amish oral health problems to eliminate selection bias so that we can understand the dynamics of their oral health care.
